# Supplementary material for: End Sequence Analysis Toolkit (ESAT) expands the extractable information from single-cell RNA-seq data
Source: Genome Res. 2016 Oct;26(10):1397–410. doi: 10.1101/gr.207902.116 (PMC5052061; doi:10.1101/gr.207902.116)
Supplement: Supplemental Material [file supp_gr.207902.116_Supp_Materials.tar.gz › ESAT-esat_v0.1/doc/html/classbroad_1_1core_1_1parser_1_1_string_parser.html]

ESAT: broad.core.parser.StringParser Class Reference


ESAT


- Main Page
- Related Pages
- Packages
- Classes
- Files

- Class List
- Class Index
- Class Hierarchy
- Class Members

All Classes Namespaces Files Functions Variables Enumerator Pages

- broad
- core
- parser
- StringParser

Public Member Functions |
Static Public Member Functions |
List of all members

broad.core.parser.StringParser Class Reference

|  |  |
| --- | --- |
| Public Member Functions | |
|  | StringParser () |
|  | |
| void | parse (String s) |
|  | |
| void | clear () |
|  | |
| void | parse (String s, String regexp) |
|  | |
| int | getIndexFor (String string) |
|  | |
| int | getFieldCount () |
|  | |
| String | asString (int index) |
|  | |
| int | asInt (int index) |
|  | |
| long | asLong (int index) |
|  | |
| double | asDouble (int index) |
|  | |
| float | asFloat (int index) |
|  | |
| String[] | getStringArray () |
|  | |

|  |  |
| --- | --- |
| Static Public Member Functions | |
| static String | firstField (String s) |
|  | |
| static String[] | getTokens (String s) |
|  | |

## Detailed Description

Author
:   prussell Parse a string around whitespace or another specified delimiter Get full array of parsed strings or individual values by index, parsed to various types

## Constructor & Destructor Documentation

|  |  |  |  |  |
| --- | --- | --- | --- | --- |
| broad.core.parser.StringParser.StringParser | ( |  | ) |  |

Here is the caller graph for this function:

## Member Function Documentation

|  |  |  |  |  |  |
| --- | --- | --- | --- | --- | --- |
| double broad.core.parser.StringParser.asDouble | ( | int | *index* | ) |  |

Gets the token at specified position and parses to a double

Parameters
:   |  |  |
    | --- | --- |
    | index | The position |

Returns
:   the desired token as a double

|  |  |  |  |  |  |
| --- | --- | --- | --- | --- | --- |
| float broad.core.parser.StringParser.asFloat | ( | int | *index* | ) |  |

Gets the token at specified position and parses to a float

Parameters
:   |  |  |
    | --- | --- |
    | index | The position |

Returns
:   the desired token as a float

|  |  |  |  |  |  |
| --- | --- | --- | --- | --- | --- |
| int broad.core.parser.StringParser.asInt | ( | int | *index* | ) |  |

Gets the token at specified position and parses to an int

Parameters
:   |  |  |
    | --- | --- |
    | index | The position |

Returns
:   the desired token as an int

|  |  |  |  |  |  |
| --- | --- | --- | --- | --- | --- |
| long broad.core.parser.StringParser.asLong | ( | int | *index* | ) |  |

Gets the token at specified position and parses to a long

Parameters
:   |  |  |
    | --- | --- |
    | index | The position |

Returns
:   the desired token as an int

|  |  |  |  |  |  |
| --- | --- | --- | --- | --- | --- |
| String broad.core.parser.StringParser.asString | ( | int | *index* | ) |  |

Gets the token at specified position

Parameters
:   |  |  |
    | --- | --- |
    | index | The position |

Returns
:   the desired token as a String

Here is the caller graph for this function:

|  |  |  |  |  |
| --- | --- | --- | --- | --- |
| void broad.core.parser.StringParser.clear | ( |  | ) |  |

|  |  |  |  |  |  |
| --- | --- | --- | --- | --- | --- |
| static String broad.core.parser.StringParser.firstField | ( | String | *s* | ) |  |

static

Get the first field of a whitespace-delimited string

Parameters
:   |  |  |
    | --- | --- |
    | s | The string |

Returns
:   The first field

Here is the call graph for this function:

|  |  |  |  |  |
| --- | --- | --- | --- | --- |
| int broad.core.parser.StringParser.getFieldCount | ( |  | ) |  |

Get number of fields

Returns
:   number of fields

Here is the caller graph for this function:

|  |  |  |  |  |  |
| --- | --- | --- | --- | --- | --- |
| int broad.core.parser.StringParser.getIndexFor | ( | String | *string* | ) |  |

Get position of the string in array

Parameters
:   |  |  |
    | --- | --- |
    | string | The string |

Returns
:   The first position equal to the string

|  |  |  |  |  |
| --- | --- | --- | --- | --- |
| String [] broad.core.parser.StringParser.getStringArray | ( |  | ) |  |

Gets the array of tokens

Returns
:   all the parsed tokens as a String[]

|  |  |  |  |  |  |
| --- | --- | --- | --- | --- | --- |
| static String [] broad.core.parser.StringParser.getTokens | ( | String | *s* | ) |  |

static

Parses the string around whitespace and stores tokens

Parameters
:   |  |  |
    | --- | --- |
    | s | The string to parse |

Returns
:   Tokens

|  |  |  |  |  |  |
| --- | --- | --- | --- | --- | --- |
| void broad.core.parser.StringParser.parse | ( | String | *s* | ) |  |

Parses the string around whitespace and stores tokens

Parameters
:   |  |  |
    | --- | --- |
    | s | The string to parse |

|  |  |  |  |
| --- | --- | --- | --- |
| void broad.core.parser.StringParser.parse | ( | String | *s*, |
|  |  | String | *regexp* |
|  | ) |  |  |

Parses the string around the specified delimiter and stores tokens

Parameters
:   |  |  |
    | --- | --- |
    | s | The string to parse |
    | regexp | Regular expression for delimiter (for period use "\\.") |

---

The documentation for this class was generated from the following file:

- java/broad/core/parser/StringParser.java


---

Generated on Fri Aug 15 2014 15:10:55 for ESAT by  

 1.8.7
